# Development of a text message intervention aimed at reducing alcohol-related harm in patients admitted to hospital as a result of injury

**DOI:** 10.1186/s12889-015-2130-6

**Published:** 2015-08-22

**Authors:** Sarah Sharpe, Matthew Shepherd, Bridget Kool, Robyn Whittaker, Vili Nosa, Enid Dorey, Susanna Galea, Papaarangi Reid, Shanthi Ameratunga

**Affiliations:** Section of Epidemiology and Biostatistics, School of Population Health, University of Auckland, Private Bag 92019, Auckland, 1142 New Zealand; School of Counselling, Human Services and Social Work, University of Auckland, Auckland, New Zealand; National Institute for Health Innovation, University of Auckland, and Waitemata District Health Board, Auckland, New Zealand; Pacific Health Section, School of Population Health, University of Auckland, Auckland, New Zealand; The National Institute for Health Innovation, University of Auckland, Auckland, New Zealand; Community Alcohol & Drug Services, Auckland, New Zealand; Waitemata District Health Board, Auckland, New Zealand; Centre for Addictions Research & Honorary Senior Lecturer, University of Auckland, Auckland, New Zealand; Te Kupenga Hauora Māori, Faculty of Medical & Health Sciences, University of Auckland, Auckland, New Zealand

## Abstract

**Background:**

Screening for alcohol misuse and brief interventions (BIs) for harm in trauma care settings are known to reduce alcohol intake and injury recidivism, but are rarely implemented. We created the content for a mobile phone text message BI service to reduce harmful drinking among patients admitted to hospital following an injury who screen positive for hazardous alcohol use. The aim of this study was to pre-test and refine the text message content using a robust contextualisation process ahead of its formal evaluation in a randomised controlled trial.

**Methods:**

Pre-testing was conducted in two phases. First, in-depth interviews were conducted with 14 trauma inpatients (16–60 years) at Auckland City Hospital and five key informants. Participants were interviewed face-to-face using a semi-structured interview guide. Topics explored included: opinions on text message ideas and wording, which messages did or did not work well and why, interactivity of the intervention, cultural relevance of messages, and tone of the content. In a second phase, consultation was undertaken with Māori (New Zealand’s indigenous population) and Pacific groups to explore the relevance and appropriateness of the text message content for Māori and Pacific audiences.

**Results:**

Factors identified as important for ensuring the text message content was engaging, relevant, and useful for recipients were: reducing the complexity of message content and structure; increasing the interactive functionality of the text message programme; ensuring an empowering tone to text messages; and optimising the appropriateness and relevance of text messages for Māori and Pacific people. The final version of the intervention (named ‘YourCall^™^’) had three pathways for people to choose between: 1) text messages in English with Te Reo (Māori language) words of welcome and encouragement, 2) text messages in Te Reo Māori, and 3) text messages in English (with an option to receive a greeting in Samoan, Tongan, Cook Island Māori, Niuean, Tokelauan, Tuvaluan, or Fijian).

**Conclusions:**

We have developed a text message intervention underpinned by established BI evidence and behaviour change theory and refined based on feedback and consultation. The next step is evaluation of the intervention in a randomised-controlled trial.

## Background

Injury is the largest contributor to New Zealand’s alcohol-related burden of disease, [1–3] and alcohol is considered the leading risk factor for injury [4–7]. One in three New Zealanders who consume alcohol have reported being harmed by their own drinking in the past year [8]. Factors associated with a higher risk of alcohol-related harm were being male, younger age, Māori ethnicity (New Zealand’s indigenous population), or living in a very deprived area of New Zealand [8].

Screening and BI is an important component of a comprehensive public health strategy to reduce hazardous alcohol use and prevent alcohol-related harm. A large body of evidence has established the effectiveness of screening and BI in a wide range of health-care settings [9–13]. For injured patients attended to in trauma care settings, BI can reduce subsequent alcohol intake and alcohol-related harms [14]. In a systematic review of BI studies for injury patients, Nilsen and colleagues concluded that, although it was difficult to provide evidence on the results of BI due to heterogeneity of studies, 11 of the 12 studies that compared pre- and post-BI results observed a significant effect of BI on at least some of the outcomes of interest (alcohol intake, risky drinking practices, alcohol-related negative consequences, and injury frequency) [14].

In New Zealand, however, screening and BI is infrequently implemented in trauma care settings. A retrospective analysis of trauma registry data (*n* = 1970) and hospital records (*n* = 120) of adults aged ≥18 years with unintentional injury admitted to Auckland City Hospital, a tertiary-level metropolitan trauma centre, reported that none of the 120 records reviewed had documentation indicating a structured questionnaire-based alcohol screening had been conducted and just one patient was recorded as having received a brief alcohol intervention. This was despite 23 % of patient records containing documentation indicating problem drinking and/or evidence of alcohol consumption prior to injury [15]. Similar findings of low uptake are also reported in the United States despite the recommendation that screening and BI is incorporated as a routine component of trauma care [16, 17]. A USA national survey of Emergency Department (ED) directors at Level I and Level II trauma centres found that, of the 46 % who responded to the survey, only 15 % reported having formal screening and intervention policies in their ED [16]. Previous research has indicated a range of barriers to implementation, including lack of resources and training of health professionals [15, 16, 18, 19].

The use of mobile (cellular) phones as the mode of BI delivery could address some of these barriers. Communicating via text message is cost-effective, highly scalable, and has the potential to reduce inequities in access to health promotion messages and services [20, 21]. Because of the high uptake of mobile phones globally, the reach of mobile health (mHealth) interventions could be extensive. Mobile phone uptake is high among Māori and Pacific peoples in New Zealand. In the New Zealand 2013 Census, access to mobile phones within households was 86 % for Māori, 85 % for Pacific Peoples, and 87 % for the total population. In contrast access to telephone and the Internet were lower for Māori (72 and 67 %) and Pacific Peoples (77 and 65 %) than the total population (87 and 82 %) [22].

Text messages, which are by definition short in length, could be a particularly appropriate mechanism for delivering BI for hazardous drinking, as suggested by three small feasibility studies published previously. Suffoletto & colleagues (2011) demonstrated the potential of a BI via text message to reduce harmful drinking in a randomised controlled trial among 45 hazardous drinkers aged 18 to 24 years seen in three urban EDs in Western Pennsylvania, USA [23]. In a randomised controlled feasibility study in Dundee, UK, Crombie & colleagues found that a text message BI could engage participants, disadvantaged men aged 25 to 44 years who were recruited through primary care and community outreach, and had the potential to modify their binge-drinking behaviour [24]. In a qualitative study of 30 trauma inpatients aged ≥16 years in Auckland, New Zealand, Kool et al. found that the majority of participants supported the idea of a text message intervention for hazardous drinking [25]. This study found that receptiveness to messages would be increased if messages were non-judgemental and supportive, evidence-based, informative (e.g. information on the consequences of drinking and providing practical advice), and culturally relevant for Māori. Participants in this feasibility study noted the importance of ensuring the messages were not delivered too frequently and the need to be mindful of avoiding a sense of invasion of privacy and confidentiality.

More recently, Suffoletto & colleagues (2014) have reported the findings of a large 3-arm randomised controlled clinical trial of a 12-week text message alcohol intervention for ED patients aged 18 to 25 years, carried out at four urban hospitals in Pittsburgh, Pennsylvania [26]. Patients reporting hazardous alcohol consumption on screening were eligible to participate and were randomised to one of three groups: text message intervention involving assessments and feedback (*n* = 384), text message assessments only (*n* = 196), no text messages (control, *n* = 185). At 3-months follow-up, the intervention group showed small reductions from baseline in self-reported binge-drinking days (intervention group: −0.51; 95 % Confidence Interval (CI) −0.10,−0.95, cf. assessment group: 0.90; 95 % CI 0.23, 1.6, and control group: 0.41; 95 % CI −0.20,1.0) and the number of drinks consumed per drinking day (intervention group: −0.31; 95 % CI −0.07, −0.55, cf. assessment group: 0.10; 95 % CI −0.27, 0.47 and control group: 0.39; 95 % CI 0.06, 0.72).

Whilst there is a large body of literature pertaining to alcohol BIs in trauma care settings, there are just the four studies outlined above which explore mHealth alcohol BIs, with just Kool et al. being specifically focussed on the trauma inpatient setting, and none which focus on developing and testing content which is culturally appropriate for an indigenous population. Building on the information noted by Kool et al. as the foundation, our research group formulated the concept of a proactive, automated mobile phone text message BI service aimed at reducing hazardous drinking and alcohol-related harm among adults admitted to hospital following an injury and who screen positive for alcohol misuse. Our plan was to tailor the text message content to suit different demographic groups (e.g. age, gender, and Māori and Pacific ethnic groups), and design the delivery of the BI to be resource-efficient, accessible to youth and socio-economically disadvantaged groups, and scalable nationwide.

We assembled an Intervention Development Team (the authors of this paper) to oversee and guide the development of the text message intervention. The team was comprised of experts in mobile phone health technology, drug and alcohol clinical services, clinical and health psychology, public health, youth health, and Māori, Pacific, and Asian health. The group discussed and developed the intervention concept, created the initial text message content, reviewed findings from pre-testing, and made decisions about refinements of text message content and structure.

This aim of this study was to pre-test the text message BI content so that the content could be improved and refined to enhance its acceptability and potential effectiveness in the local context. It was intended that the effectiveness of the refined intervention in reducing harmful drinking would subsequently be evaluated in a randomised-controlled trial.

## Methods

**Fig. 1:**
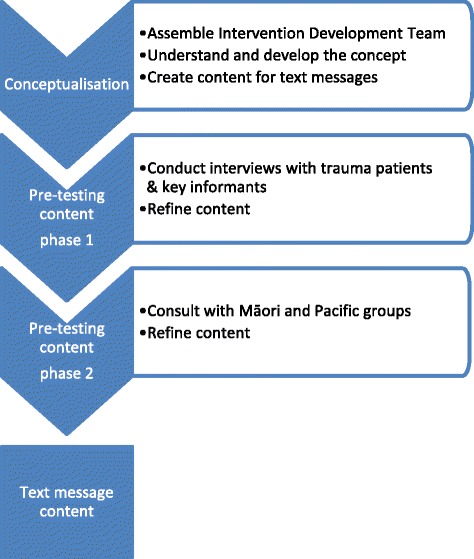
Process for text message intervention content development

Our methodologic approach is described in Fig. 1 and was informed by Whittaker and colleagues’ model for developing and evaluating mHealth interventions [27]. This model describes a process in which the intervention created is based on theory and evidence, the target audience is involved to ensure the intervention is engaging and useful, and there is a focus on implementation from the outset.

The initial text message content was created based on the BI model [28] and the Stages of Change behaviour change theory [29] which underpins the model. BI has three key steps: 1) giving feedback and information about a person’s current behaviour (in this case, hazardous alcohol use), 2) listening and discussing the issue, and 3) giving advice, discussing options, and helping with goal-setting [17, 28, 30]. The Development Team wanted to mimic the underlying key BI elements as far as possible, whilst being mindful of the limitation that text messaging lacks the face-to-face interpersonal component of conventional BI. Using Microsoft Excel, we mapped the key elements of BI (as described by Babor & Higgins-Biddle [28]) and recommendations for its use in a trauma setting (the American College of Surgeons Committee on Trauma [17]) against a variety of behaviour change techniques and crafted short messages for each of the key elements (Table 1). Readability testing showed the content of these messages had a Flesch-Reading Ease score of 66.6 and Flesch-Kincaid Grade Level score of 6.6.

**Table 1:** Original text message content

Message number	Week of programme	Day of programme	Text message	Brief Intervention element	Behaviour change techniques
1	1	1	Thanks 4 joining the study. Txt messages will be coming 2 your mobile over the next 4 weeks. Call xxx free if you have any study-related problems		
2	1	2	The survey showed your alcohol drinking is hazardous compared with other people. We recommend you think about cutting down	Feedback about screening results; recommendation	Feedback; comparison/discrepancy
3	1	4	*For females:*	Information on drinking limits	Information
			Recommended drinking limit for females = max 2 drinks/day and max 10 drinks/week. 1 drink = 100mls wine or 330mls beer or 30mls spirits or half a premix (RTD)		
			*For males:*		
			Recommended drinking limit for males = max 3 drinks/day and max 15 drinks/week. 1 drink = 100mls wine or 330mls beer or 30mls spirits or half a premix (RTD)		
4	1	6	Alcohol can cause injuries, diseases like cancer, depression, weight gain…plus hangovers are awful! Make a list of the pros and cons of drinking too much alcohol	Information on hazards of drinking; encourage/motivate RTC	Information on consequences; Persuasive communication; motivators for change; pros and cons
5	2	8	Would you be willing to make changes to reduce your drinking? If your answer is 'Yes', txt 1 to yyy. If your answer is 'No', txt 2 to zzz	Assess RTC	Assessment; Support/encourage change; Prompt intention formation
If answer ‘Yes’ to Message ID 5:					
6	2	10	Great news that you are willing to reduce your alcohol use! Keep your reasons in mind. We would like to help and will txt you tips and advice	Encourage/motivate RTC	Support/encouragement
7	2	11	*For females:*		
			Consider setting a goal to reduce drinking to within safe limits: max 2 drinks/day, max 10 drinks/week. (1 drink = 100 ml wine or 330 ml beer or 30 ml spirits)	Advice based on RTC 'Yes': Goal	Goal-setting
			*For males:*		
			Consider setting a goal to reduce drinking to within safe limits: max 3 drinks/day, max 15 drinks/week. (1 drink = 100 ml wine or 330 ml beer or 30 ml spirits)		
8	3	15	Plan ahead 4 cutting down your alcohol use. Consider setting some drinking rules for yourself. See easeuponthedrink.org.nz for more info	Advice based on RTC 'Yes': Plan	Planning; Coping strategies; prompt self-monitoring
9	3	17	Consider sharing your goal and plan with friends and family. They can provide support and might want to join in and reduce their alcohol drinking too	Advice based on RTC 'Yes': Strategies/tips	Coping strategies; Support/encouragement
10	3	19	Ideas for helping you cut down: consider planning alc-free days, measure and track drinks, alternate alc and non-alc drinks, avoid risky circumstances	Advice based on RTC 'Yes': Strategies/tips	Coping strategies; Support/encouragement
11	4	22	Reward yourself 4 your successes. Learn from slip-ups but don't dwell on them. Don't give up on your goal to reduce drinking!	Advice based on RTC 'Yes': further support	Support/encouragement; review of goal; self-reward; relapse prevention
12	4	24	You can get more free and confidential support from Alcohol Helpline 0800 787 797, or by contacting your family doctor	Follow-up	Information; Support/encouragement
13	4	27	Thanks for taking part in the study. We will txt u in 2 months to see how u r going.		
If answer ‘No’ to Message ID 5:					
14	2	10	Thanks 4 your reply. Drinking alcohol is your choice. Txts to follow about ways to minimise harm from alcohol	Advice based on RTC 'No'	Support; Coping strategies
15	2	11	Reduce your risk of injury on a single occasion of drinking by setting a limit of no more than 4 standard drinks. (1 drink = 100 ml wine or 330 ml beer or 30 ml spirits)	Advice based on RTC 'No'	Information; Coping strategies
16	3	15	Consider planning alc-free days, pacing yourself when drinking, alternating alc and non-alc drinks, taking smaller sips, eating before or while u r drinking	Advice based on RTC 'No'	Coping Strategies, Planning
17	3	17	Drinking too much alcohol can cause problems for you, your family, your friends. See easeuponthedrink.org.nz for easing up tips	Advice based on RTC 'No'	Information; Support/encouragement
18	3	19	Plan ahead so you get home safely. Arrange a designated driver. Put some cash aside and share a taxi. If you have to walk home, go with a friend	Advice based on RTC 'No'	Coping Strategies, Planning
19	4	22	We encourage you to think about your drinking. You may have bad experiences, regrets, worries. One day you may decide u want to make a change	Advice based on RTC 'No'	Support/encouragement; Prompt intention formation
12	4	24	You can get more free and confidential support from Alcohol Helpline 0800 787 797, or by contacting your family doctor	Follow-up	Information; Support/encouragement
13	4	27	Thanks for taking part in the study. We will txt u in 2 months to see how u r going.		

*RTC* = Readiness to Change

Since the time of the study, theurl easeuponthedrink.org.nz has changed to http://www.alcohol.org.nz/help-advice/ease-up-on-the-drink

We carried out a small qualitative research study to pre-test the text message content. The pre-testing was conducted in two phases: 1) interviews with trauma inpatients and key informants and 2) consultation with Māori and Pacific groups.

A purposive sampling approach was taken to ensure a mix of ethnicity groups, age groups and gender among the trauma inpatients interviewed and to ensure the views of key stakeholders were heard. The aim of the sampling strategy was to select a range of patients and key informants in order to gain insights and understanding about their perceptions regarding the content, accessibility of the messages and structure of the intervention. This study was not designed to select a statistically representative sample in order to make empirical generalisations representative of all trauma inpatients.

In the first phase of pre-testing we aimed to recruit 15 adult trauma inpatients (five Māori patients, five Pacific patients, and five patients of other ethnicities) and five key informants from the following organisations: Auckland City Trauma Service (clinical service), Alcohol HealthWatch (non-governmental agency), Accident Compensation Corporation (injury-related social insurance agency), Auckland Council (regional authority), and National Hauora Coalition (primary health organisation).

Trauma inpatient participant selection and in-depth individual interviews were carried out over a four week period during May 2012 at Auckland City Hospital. Patients could be included if they were aged 16 to 60 years, had been admitted to hospital with an injury and were under the care of the Trauma Service, used a mobile phone, were alcohol users, and could complete an interview in English. Patients were excluded if they had a cognitive deficit, a serious psychiatric disorder, or were pregnant. Potential participants were identified prospectively by daily review of the Trauma Service admission register followed by discussion with the trauma co-ordinator and/or ward staff, and then were approached in person by an interviewer and invited to take part. Three interviewers in total conducted individual face-to-face interviews, which ranged from 30 to 60 min in length. Interviews with Māori participants were conducted by a Māori researcher, and interviews with Pacific participants were conducted by a researcher who identified with Cook Island and European ethnicities. The third interviewer was New Zealand European.

All potential participants (inpatients and key informants) were provided a Participant Information Sheet and those taking part in the study gave their written informed consent. Interviews were semi-structured, with an interview guide used as an outline and prompt (Appendix 1). The questions and guide were developed by the lead authors (SS and MS), in consultation with the Development Team. Topics explored during the interviews included: opinions on text message ideas and wording (a paper-based text message prototype was provided), which messages worked well and why, which messages didn’t work well and why, interactivity of intervention, cultural relevance of messages, and tone of messages. Interviews were audio-recorded and transcribed by a commercial transcription service.

In addition to the interview, a short survey to capture basic demographic details and the participant’s Alcohol Use Disorder Test (AUDIT)-C score [31] was administered. Ethnicity data was collected by using the standard ethnicity question from the New Zealand Census, as recommended by the New Zealand Ministry of Health [32]. Trauma inpatient participants received a $20 shopping voucher as a token of appreciation for taking part in the study.

The interview transcriptions were analysed at two levels: 1) by each text message, to analyse feedback and suggestions related to the content of each message, and 2) for cross-cutting themes that emerged related to content and structure of the text message intervention. A General Inductive approach was used for the second level data analysis [33]. Analyses were conducted using NVivo 9 qualitative analysis software. Interview transcripts were entered into NVivo and the raw text was examined in detail. Coding was applied to the text to indicate feedback on specific messages (level one analysis) and to indicate categories or themes (level two analysis). Within each category, the text was searched for a range of viewpoints and quotations were selected to show this range, as well as the core meaning of a theme.

Based on the findings from this small qualitative research study, the content and structure of the intervention were refined by the Intervention Development Team. Subsequently, a second phase of consultation was undertaken with Māori drug and alcohol counsellors (Te Ātea Marinō), Pacific drug and alcohol counsellors (Tupu), Pacific staff at the University of Auckland, and Māori researchers, to enhance the relevance, appropriateness and acceptability of the text message intervention content to Māori and Pacific communities.

Ethical approval for the qualitative research component was obtained from the Northern X Regional Ethics Committee (NTX/11/EXP/307), the Auckland District Health Board, and the Waitemata District Health Board.

## Results

Nineteen trauma inpatients were approached, 14 were interviewed, and five declined or were not eligible due to not being drinkers. Participants ranged in age from 17 to 50 years and the majority were male (Table 2). Four participants identified as New Zealand Māori. One of these participants also identified as Cook Island Māori and another as Niuean. Three participants identified as Samoan, five were European, and two were Asian (Chinese and Filipino). AUDIT-C scores ranged from 0 to 9 with a median of 5. Nine participants were categorised as having a pattern of drinking considered hazardous (AUDIT-C score ≥3 for women and ≥4 for men). Injuries sustained by participants included limb fractures or lacerations (*n* = 6), head injuries (*n* = 4), chest injuries (*n* = 2), fractured pelvis (*n* = 1), fractured lumbar vertebra (*n* = 1). Alcohol was a contributing factor in four cases.

**Table 2:** Characteristics of trauma inpatients (*n* = 14)

Characteristic	Number of participants
Gender	
Male	11
Female	3
Age group	
16 – 34 years	9
35 – 54 years	5
Ethnic group	
Māori	4^a^
Pacific Peoples	5^a^
European	5
Asian	2
Employment status	
Employed	7
Student	5
Unemployed or Other	2
AUDIT-C score indicating hazardous drinking^b^	
Non-hazardous drinking	5
Hazardous drinking	9

^a^The ethnic data in this table is reported using the total response (overlapping) method. Where a person reported more than one ethnic group, that individual has been counted in each applicable group. Totals therefore do not add up to 100 percent. One participant identified as New Zealand Māori and Cook Island Māori. Another participant identified as New Zealand Māori and Niuean

^b^AUDIT-C is scored on a scale of 0–12; in men a score of 4 or more, and in women a score of 3 or more, is considered positive for identifying hazardous drinking or active alcohol use disorders

### Phase one: Feedback on specific text messages

Participants provided many suggestions for improvements to the content of each text message (Table 1). A commonly-expressed issue was specific words not being easily understood or relevant to people. For example, the use of the word ‘hazardous’ in message two (*‘The survey showed your alcohol drinking is hazardous compared with other people. We recommend you think about cutting down’*) was not felt to be appropriate by the majority of participants:

*“Hazardous is a bit of a big word for some folks isn’t it. Usually hazardous you think of bombs and explosions.” (Male, New Zealand European inpatient, non-hazardous drinker)*

However, one participant liked the direct nature of this message:

*“I think that’s really good because then it kind of makes people think maybe I’m hurting other people or hurting myself.” (Female, Filipino inpatient, hazardous drinker)*

The information-laden content (summarising low risk drinking guidelines and the definition of a ‘standard’ drink) of text message three positioned at this early stage of the intervention was generally perceived to be negative and off-putting:

*“Well there’s quite a bit of information there, most people probably turn it off at that point. Well the problem with text messages you know, texts are usually some social thing,.…” (Male, New Zealand European inpatient, non-hazardous drinker)*

One respondent aged in his early 20s indicated that this kind of message would be meaningless for him and his friends: *“I think people our age would laugh. I don’t think they’d take any notice of it to be honest, … So you don’t drink just to drink, you drink to get drunk, that sounds really bad but that’s how it is.” (Male, Samoan inpatient, hazardous drinker)*

In addition, we were advised by a key informant that the use of the words ‘recommended drinking limits’ was not a helpful approach.

*“So a limit’s actually meaningless. What language …we’re trying to find ways of sharing I suppose, is the risk, levels of risk and low risk, you know beyond two standard drinks you are at higher risk. So it’s not about trying to set a limit …you know it’s like a speed limit, people treat it as a target, they think I can drive 100… in actual fact they should be thinking about the conditions and is it wet and perhaps I should be driving you know 80 today on this road…So we’re trying to get people away from the idea of limits.” (Key informant)*

Many participants noted that instead of receiving this information message during the first week of the text message intervention, they would rather receive a message that was linking them into existing services and advice. In the prototype, such a message (number 12) was planned for the fourth week. This was perceived to be too late in the intervention, particularly for people who might feel anxious after receiving the message or who might want to take prompt action and seek help based on information provided early in the intervention process. Many participants liked the idea of having a website link in addition to a free-call number for the Alcohol Helpline.

A key element of BI is motivating and encouraging people to change their behaviour. Behaviour change techniques include providing information on consequences, using persuasive communication, discussing motivators for change, and thinking about the pros and cons of drinking alcohol. We incorporated some of these ideas in text message number four, with the aim of encouraging people to contemplate their drinking, the effects of drinking, and stimulate readiness to change. In general, participants liked this text message. They were interested in thinking about pros and cons and the effects of alcohol, and in many cases displayed a lack of knowledge about alcohol and its effects on the body.

*“… I like that it actually gives you something you can do on your own, how it’s like suggest that you make a list of pros and cons. It’s kind of like a tool you can use so that’s quite helpful.” (Female, Samoan inpatient, hazardous drinker)*

*“It’s got a little bit of information in it and it also makes you think. If you did a list yourself for or against, you know, pros and cons, …you know there’s more negative stuff to it than there is positive.” (Male, New Zealand European inpatient, hazardous drinker)*

It was clear that there were a wide range of motivators and it would be very difficult to create one message that would appeal to people of different age, gender, and ethnicity groups. However, many participants talked about the effect of alcohol consumption not just on themselves, but on their family/whānau and friends, suggesting an important motivator in our communities.

*“…You have got to find what’s going to motivate each individual person in a way, and it’s going to vary, person to person…so that’s the tricky part isn’t it and I can imagine some people at this point will just turn off, you know the negative sort of message about the cancer, weight gain, that sounds bad and better go and have a drink.” (Male, New Zealand European inpatient, non-hazardous drinker)*

*“… is it something more about …, the effect of too much drinking on your friends and family or something.” (Male, New Zealand European inpatient, non-hazardous drinker)*

*“Family, there’s always a family issue, it’s stressing when someone ends up here [i.e. in hospital].” (Male, Māori/Niuean inpatient, hazardous drinker)*

Following on from the ‘motivator’ text message, text message number five (at the beginning of week two of the text message intervention) was proposed to be a question evaluating a person’s readiness to change [30, 34]. The intervention would branch at this point, with those responding ‘Yes’ receiving text message relevant to making changes, and with those responding ‘No’ receiving supporting messages providing information and encouraging contemplation about drinking alcohol. Many participants didn’t like this message, as highlighted in these next quotes. These feelings may be due to participants not being comfortable committing to a goal which has been assigned by others, is framed in a way that is not desirable to the participant, and is not linked with any specific contexts or strategies (*i.e.* implementation intentions) that might help with goal striving [35, 36].

*“Me personally I wouldn’t like that one because I’ve only just started on this, haven’t really had much time to think about things. It might be a bit too soon.” (Male, New Zealand European inpatient, hazardous drinker)*

*“I’m debating about, because you’ve got if you’re willing to make changes, yes or no. If you’ve answered ‘no’ and you are still getting texts coming through…Yeah, might make them annoyed.” (Key informant)*

*“I was just thinking you’re asking questions and then they’re asked to provide a response but they’re not told why? So I would think well what’s the point of replying…it’s not giving me any reason to do that. What may happen if I do that?” (Female, New Zealand European inpatient, non-hazardous drinker)*

### Phase one: Cross-cutting themes

Responses from participants were also explored for themes that cut across all text messages. There were four main themes related to reducing the complexity of message content and intervention structure, increasing the interactivity of the intervention, ensuring an empowering tone to text messages, and optimising cultural appropriateness and relevance.

### Theme one: Complexity of message content and structure

It was clear that the message content, as originally designed, was too complex, contained too much health/technical jargon and was not focussed adequately on reducing health literacy demands on people.

*“One thing that I learnt about text messaging stuff, people switch off after too many words.” (Key informant)*

*“Yeah, you don’t really want to read too much.” (Female, Filipino inpatient, hazardous drinker)*

*“Because I mean the health language doesn’t, you know, it’s not, it just doesn’t gel…Is it a bit medical, is it a bit sort of medicalised?” (Key informant)*

*“I think you have got to road test the language…the language is pretty complex for most people.” (Male, New Zealand European inpatient, non-hazardous drinker)*

In relation to the use of text language and abbreviations, most participants like to have words spelt in full, but were happy with short abbreviations such as ‘you’ abbreviated to ‘u’ and ‘for’ abbreviated to ‘4’.

*“Actually like English is my second language so I just like text fully, full English.” (Male, Chinese inpatient, non-hazardous drinker)*

*“I prefer it when people don’t use text language because it is too hard to read sometimes.” (Female, Filipino inpatient, hazardous drinker)*

There was positive feedback about the proposed length of the intervention and the frequency of receiving text messages. Most respondents thought the prototype’s four week length and frequency of one text message every two days were reasonable and appropriate.

*“.. that’s reasonable…definitely long enough.” (Male, New Zealand European inpatient, hazardous drinker)*

*“I think that’s good yes, because people’s interest loses after sort of six weeks or so, so probably four weeks is a good timeframe.” (Key informant)*

*“If you are getting a text every day then you might start to ignore it, but if you get one every couple of days it might get through actually…” (Male, New Zealand European inpatient, hazardous drinker)*

### Theme two: Interactive functionality of the text message programme

The prototype was designed as an automated and unidirectional text message intervention, although we attempted to personalise content to some extent. This was well received although some participants voiced a desire for the intervention to have more interactive functions, so that they could text back and forth with someone.

*“You have definitely got some good ideas here. I think maybe some of them text back so you know that they are getting through? Maybe a text back to every one of these… so you know they are getting through.” (Male, New Zealand European inpatient, hazardous drinker)*

*“You’re more likely to be honest or take more notice if you think there is somebody at the other end that sent me this text, not a machine that sent me that text. I think you would get a better response because people then feel, oh someone is making all this effort I should make an effort as well, rather than, like you get texts from x [a mobile phone provider] or y [a mobile phone provider] and it’s automated, and you go oh yeah, whatever.” (Key informant)*

A small number of participants said automation wasn’t a negative aspect for them and that they appreciated the anonymous nature of the text message intervention.

*“… Pacific Island culture in general it’s like there are a lot of things that you don’t talk about… so I think people would sign up for this because it seems like something you can do personally that you don’t have to tell people about. So you don’t have to talk about it…I think getting the texts would be helpful cause then it wold be like a way for you to kind of like reflect and then like cut down.” (Female, Samoan inpatient, hazardous drinker)*

### Theme three: Tone of messages

The importance of the tone of the messages to be empowering and encouraging, and not in any way condescending or laying blame, was a common feature in the feedback received.

*“People could take it one or two ways. They could agree with it or they could feel as though they could be being judged in some way and they don’t even know the people who are really judging them, saying that. Some people could get offended and some people might not.” (Male, New Zealand European inpatient, hazardous drinker)*

*“It just seems more approachable if you’re saying that like you recommend it instead of you should cut down bla bla bla…I quite like the tone of it…Cause it kind of makes you like reflect. And it doesn’t seem too direct, you have to think about your drinking, it’s just real like it would be a good idea, it seems more helpful. As opposed to, like, confrontation.” (Female, Samoan inpatient, hazardous drinker)*

### Theme four: Language alignment

Māori, Pacific, and Asian participants were specifically asked what they thought about having greetings in their own language in the content of text messages. The majority responded that they thought this would be a good idea to help make the intervention more personalised and engaging.

*“Different greetings…. Because it’s just the sense of them knowing who you are and where you’re from. They’ve done the research in terms of understanding what ethnic background you are.” (Male, Samoan inpatient, hazardous drinker)*

*“It should be just Kia ora because not all Māori can speak Te Reo.” (Male, Māori/Niuean inpatient, hazardous drinker)*

For many participants, acknowledging the important role of family/whānau in their lives was central to the intervention content, particularly the ‘motivator’ text message to be delivered during week one of the intervention. For Māori participants, not only was the concept of whānau important, but also utilisation of whānau, the Te Reo (Māori language) word.

*“I think a lot of things when they are done in a family sort of setting, you know, maybe work better.” (Male, Māori inpatient, hazardous drinker)*

*“.. whānau is real, everybody know that word…it could personalise it a bit…appeal to them more.” (Male Māori/Niuean inpatient, hazardous drinker)*

There was support from participants for a Te Reo Māori translation of the text messages. But it was also seen to be important to have the choice of an English version with some relevant Te Reo Māori words incorporated, as participants said there were many Māori who were not fluent in Te Reo Māori.

*“It might have more meaning for some people if it is in Te Reo, they might feel more responsible I guess.” (Key informant)*

*“… what we have to keep in mind in the population is that while there are fluent speakers of Te Reo, there are also many Māori that aren’t so there are key words like greetings, whānau is definitely a word that is used by non-Māori as well but I think it is keeping that in mind or having the option for a translated version as well but they choose that option.” (Key informant)*

### Phase two: Consultation with Māori and Pacific groups

Following revision and refinement of the text message intervention prototype based on findings from the first phase of pre-testing described above, consultation with Māori and Pacific groups was undertaken. Further refinements included reducing the number and length of text messages, changing content to be more relevant for Māori and Pacific audiences (e.g. inserting Te Reo Māori words of encouragement in appropriate places, changing specific words if the meaning was not clear), and translating the text messages to Te Reo Māori.

During this phase, the name of the text message intervention was considered. We received substantial feedback during the pre-testing phase and consultation with Māori and Pacific groups that the initial name ‘MoDeRATE’ (M-health Delivery for Reducing Alcohol in the Trauma Environment Trial) was unappealing, not engaging nor empowering, and lacked meaning. For many people, ‘moderation’ in relation to drinking was not a familiar concept. We engaged an advertising agency to help come up with a new name. We trialled three options and chose the name ‘YourCall’. This name was seen to be positive, inspiring, and represented a challenge or ‘call to action’.

### Final version of text message intervention

The final version of the text message intervention had three main language-based text message pathways for people to choose between: 1) text messages in English with Te Reo Māori words of welcome and encouragement, 2) text messages in Te Reo Māori, and 3) text messages in English (with an option to receive a greeting in Samoan, Tongan, Cook Island Māori, Niuean, Tokelauan, Tuvaluan, or Fijian).

The finalised structure of the text message intervention was less complex compared with the original prototype. It consisted of 16 text messages in total spread over a one month period (Table 3). Single messages were to be sent at two-day intervals, with two exceptions, when two related messages were to be sent in tandem, half an hour apart. Messaging was designed to commence on a Monday and finish on a Saturday. Text messages falling on a week-day were to be sent at 7 pm and those falling on a weekend-day were to be sent at 3 pm.

**Table 3:** YourCall text message intervention content

Week of programme	Day of programme	English^a^	English with some Te Reo Māori words	Te Reo Māori
1	1 (Mon)	From YourCall: Hi, thanks 4 taking part in the study. Over the next 4 weeks we will be sending u txts with info & ideas	From YourCall: Tena koe. Thanks 4 taking part in the study. Over the next 4 weeks we will be sending u txts with info & ideas	Mai i TōWaea: Tēnā koe. Ngā mihi ki a koe i whai wāhi mai ki te rangahautanga. Mō te 4 wiki e tū mai nei ka tuku kupu kawe pārongo, kawe whakaaro ki a koe
1	3 (Wed)	YourCall: Your survey responses show your drinking is harmful 2 your health. Make a positive change in your life – cut down or quit	YourCall: Kia ora. Your survey responses show your drinking is not good 4 your health and wairua. Make a positive change in your life – cut down or quit	TōWaea: Kia ora. Nā ō whakautu rangahau kua kitea kāore e pai te waipiro ki tō hauora, wairua hoki. Tahuri ki te pai hei oranga mōu–whakaitia, whakamutua rānei
1	3 (Wed)	YourCall: U can get confidential support from Alcohol Helpline ph 0800 787 797 web alcoholdrughelp.org.nz or your doctor	YourCall: Kia ora. U can get confidential support from Alcohol Helpline ph 0800 787 798 web alcoholdrughelp.org.nz or your doctor	TōWaea: Kia ora. Ka taea te tautoko matatapu mai i te Alcohol Helpline waea 0800 787 798, ipurangi alcoholdrughelp.org.nz, mai i tō tākuta rānei
1	5 (Fri)	YourCall: Alcohol may be causing problems for u, your family & friends. We encourage u 2 think about your drinking and its impact on your life	YourCall: Kia ora. Alcohol may be causing problems for u, your whānau & friends. We encourage u 2 think about your drinking and its impact on your life & whānau	TōWaea: Kia ora. Kei te whakararu pea te waipiro i a koe, i tō whānau, i ō hoa hoki. Tēnā, āta whakaarohia tō inuinu me ana pānga ki a koe, oti rā ki te whānau
1	7 (Sun)	YourCall: U might find it helpful 2 think about the good things & the not so good things about your drinking. Making a list can help	YourCall: U might find it helpful 2 think about the good things & the not so good things about your drinking. Making a list can help	TōWaea: He āwhina pea ina whakaaro koe mō ngā mea pai me ngā mea kāore i te tino pai e pā ana ki tō inuinu. He āwhina anō pea tētahi rārangi
2	9 (Tues)	YourCall: We recommend u cut down or quit alcohol. Making a positive change can be hard, try small steps	YourCall: Kia ora. We recommend u cut down or quit alcohol. Making a positive change can be hard, try small steps. Kia kaha!	TōWaea: Kia ora. Ko tā mātou tūtohu me whakaiti, me mutu rānei te inu waipiro. He uaua pea te tahuringa ki te pai, iti nei, iti nei ka taea. Kia kaha!
2	11 (Thurs)	YourCall: Ideas 4 cutting down: plan no-alcohol days, have water between drinks, try low alcohol drinks like light beer. Check out easeuponthedrink.org.nz	YourCall: Kia ora. Ideas 4 cutting down: plan alcohol-free days, have water between drinks. Check out easeuponthedrink.org.nz	TōWaea: Kia ora. He whakaaro mō te whakaiti: whakaritea he rā kore inu, me inu wai i waenga i ngā inu. Tirohia easeuponthedrink.org.nz
2	13 (Sat)	YourCall: Keep track of your drinks. U could use a diary. 1 drink = 1 small bottle beer, half an RTD, half a glass wine or 1 shot spirits	YourCall: Kia ora. Keep track of your drinks. U could use a diary. 1 drink = 1 small bottle beer, half an RTD, half a glass wine or 1 shot spirits. Mauri ora	TōWaea: Kia ora. Kautehia ō inu. Whakamahia he rātaka ina hiahia. 1 te inu = 1 te pātara pia iti, he haurua RTD, he haurua wāina, 1 te inu waipiro. Mauri ora
3	15 (Mon)	YourCall: Reduce your chance of injuries & health problems by having no more than 2 drinks per day and at least 2 no-alcohol days per week	YourCall: Kia ora. Reduce your chance of injuries & health problems by having no more than 2 drinks per day and at least 2 alcohol-free days per week. Mauri ora	TōWaea: Kia ora. Whakaitihia te tūpono wharanga, rarunga hauora hoki mā te inuinu iti iho i ngā inu e 2 ia rā, me ngā rā kore-waipiro e 2 rā ia wiki. Mauri ora
3	17 (Wed)	YourCall: Think of 1 thing u can do 2 cut down your drinking. Plan ahead & take action!	YourCall: Kia ora. Think of 1 thing u can do 2 cut down your drinking. Plan ahead & take action! Kia kaha	TōWaea: Kia ora. Whakaarohia ake kotahi mahi e taea ai e koe te whakaiti tō inuinu. Whakaritea he mahere, whāia! Kia kaha
3	19 (Fri)	YourCall: Don’t drive if u have had alcohol. Arrange a sober driver, share a taxi, take a bus, walk with a friend	YourCall: Kia ora. Don’t drive if u have had alcohol. Arrange a sober driver, share a taxi, take a bus, walk with a mate	TōWaea: Kia ora. Mena kua inu koe, kaua e taraiwa waka. Whakaritea he taraiwa kore inu, hopu tekehī, hopu pahi, hīkoi tahi me tētahi hoa rānei
3	21 (Sun)	YourCall: Think about sharing your goal with friends or family. They can give u support and may also want 2 cut down	YourCall: Kia ora. Think about sharing your goal with friends or whānau. They can give u support and may also want 2 cut down. Tu meke	TōWaea: Kia ora. Whakaarotia te tiri i tō whāinga ki ō hoa, ki tō whānau rānei. Mā rātou koe e tautoko, ka hiahia whakaiti te inuinu hoki pea rātou. Tumeke
4	23 (Tues)	*For males:*	*For males:*	*For males:*
		YourCall: Its best not to drink alcohol at all if your health is not so good or u are on medication	YourCall: Its best not to drink alcohol at all if your health is not so good or u are on medication	TōWaea: He pai ake te kore inu mena kāore tō hauora i te pai, e kai pire ana rānei koe
		*For females:*	*For females:*	*For females:*
		YourCall: Its best not to drink alcohol at all if u are pregnant or might get pregnant, your health is not so good or u are on medication	YourCall: Its best not to drink alcohol at all if u are pregnant or might get pregnant, your health is not so good or u are on medication	TōWaea: He pai ake te kore inu waipiro mena kei te hapū, ka hapū pea rānei koe, kāore i te pai tō hauora, e kai pire ana rānei koe
4	25 (Thurs)	YourCall: Reward yourself 4 making progress with your goal - but not with alcohol! Don't give up on your goal, try small steps	YourCall: Kia ora. Reward yourself 4 making progress with your goal - but not with alcohol! Don't give up on your goal, try small steps. Kia kaha	TōWaea: Kia ora. Me whakanui koe i a koe anō mō te whakatata atu ki tō whāinga–engari kaua mā te waipiro! Kaua e whakarērea tō whāinga, kia āta haere. Kia kaha
4	27 (Sat)	YourCall: Remember that u can get confidential help from Alcohol Helpline 0800 787 797 or your doctor	YourCall: Kia ora. Remember that u can get confidential help from Alcohol Helpline 0800 787 798 or your doctor	TōWaea: Kia ora. Kaua e wareware ka taea te āwhina matatapu mai i Alcohol Helpline 0800 787 798, mai i tō tākuta rānei
4	28 (Sun)	YourCall: Make a positive change in your life - cut down or quit drinking alcohol. Thanks 4 taking part in the study – great effort! We'll be in touch in 2 months	YourCall: Kia ora. Make a positive change - cut down or quit drinking alcohol. Thanks 4 taking part in the study. We'll be in touch in 2 months. Kia kaha	TōWaea: Kia ora. Tahuri ki te pai – whakaitia, whakamutua rānei te inu waipiro. Ngā mihi mōu i whai wāhi mai. Hei te 2 marama ka whakapā atu anō mātou. Kia kaha

^a^The Day 1 text message has an option to receive a greeting in the following Pacific languages: Samoan, Tongan, Cook Islands, Niuean, Tokelauan, Tuvaluan, or Fijian

©Auckland UniServices Ltd, 2012

Since the time of the study, theurl easeuponthedrink.org.nz has changed to http://www.alcohol.org.nz/help-advice/ease-up-on-the-drink

The four text messages in ‘Week One’ contained content that welcomes the text message recipient, gives them feedback about their drinking, links them to existing services (e.g. free-phone alcohol help-line), and encourages contemplation about their drinking. The first text message in ‘Week Two’ contained an empathetic, yet clear recommendation to cut down on drinking. This was followed during the rest of ‘Week Two and Three’ by six messages focussed on providing information and tips/strategies about reducing alcohol consumption. The final three text messages in ‘Week Four’ contained supportive and encouraging content with the key messages re-iterated. Overall, the readability of the revised content was improved (Flesch-Reading Ease score 76.4 and Flesch-Kincaid Grade Level score 5.2).

## Discussion

This paper describes the development of appropriate (or therapeutic) content for a text message BI service aimed at reducing hazardous drinking and alcohol-related harm among trauma inpatients. Following conceptualisation and creation of initial text message content based on the BI model and behaviour change theory, the text message content was pre-tested with trauma inpatients, key informants, and Māori and Pacific groups, familiar with the setting and context for the proposed intervention. We identified four key themes that were important to ensuring the text messages were engaging, relevant, and useful for potential recipients: 1) reducing the complexity of message content and structure; 2) increasing the interactive functionality of the text message programme; 3) ensuring an empowering tone to text messages; and 4) optimising the appropriateness and relevance of text messages for Māori and Pacific people. Consultation on the latter theme with Māori and Pacific groups helped us to further improve the text messages. The final version of the ‘YourCall’ intervention had three pathways for people to choose between: 1) text messages in English with Te Reo Māori words of welcome and encouragement, 2) text messages in Te Reo Māori, and 3) text messages in English (with an option to receive a greeting in Samoan, Tongan, Cook Island Māori, Niuean, Tokelauan, Tuvaluan, or Fijian).

Our development approach and refinement of the resulting text message intervention have a number of strengths. The intervention is underpinned by established BI theory and evidence and the content development process was guided by a group of people purposively selected for their expertise in topic areas relevant to this intervention (*i.e.* drug and alcohol clinical services, mobile phone health technology, clinical and health psychology, youth health, and Māori, Pacific and Asian health). The pre-testing conducted with the target audience, key informants, and Māori and Pacific groups provides confidence that the text message content is engaging, relevant, and culturally-appropriate. The involvement of Māori and Pacific researchers was critical for being able to carry out the pre-testing in an effective and appropriate way. The resulting intervention is a proactive programme of 16 brief text messages delivered over a four week period, utilising an ‘every-day’ technology that is already integrated into people’s lives.

There are also some limitations with this research. Pre-testing the text message prototype with trauma inpatients involved a small number of participants (*n* = 14). Seven Māori and Pacific patients were interviewed, rather than the intended number of ten, as fewer than expected Māori and Pacific patients presented during the recruitment period. Although participants were selected purposively, more males (*n* = 11) than females (*n* = 3) were interviewed. This may reflect the fact that more injury inpatients are male [15]. In addition, although all participants drank alcohol (one of the inclusion criteria), five of 14 participants had AUDIT-C scores indicating non-hazardous drinking. Inclusion of these participants enabled us to explore the viewpoints of a wide range of alcohol users. Furthermore, individuals with non-hazardous usual drinking patterns could also incur injuries in the context of a drinking episode. Although a small number of inpatients were interviewed, this was supplemented by interviews with key informants and consultation with Māori and Pacific groups, including drug and alcohol counsellors. By the end of this process, we were confident we had explored and addressed a wide range of issues. More particularly, we had also reached a point where we were not gaining any new or different opinions or gleaning any significant new pieces of information, consistent with data saturation, a key attribute of rigour in a qualitative research study. While the study was not designed to yield empiric findings that are generalizable to all trauma patients, it provided rich insights regarding diverse perspectives relevant to our research objectives.

The initial feedback on the text messages from the target audience involved showing them proposed messages on paper. This is likely to be quite a different experience from receiving the messages at random times within the context of their busy and complex daily lives. However we have found this to be an important first step in the development process with members of the target audience. The development process continues to be built upon and refined [20, 37, 38]. The full participant experience of the programme (and the individual messages) within the ‘free living’ context can only be tested in a formal evaluation.

An important challenge when determining the extent to which the development of an intervention should be guided by user preferences is the possibility that content that is more consistent with preferences of users may not necessarily equate to more effective interventions. Indeed, a degree of challenge to what participants consider appealing may be necessary to prompt a realistic appraisal of the risks and harms associated with their drinking. Consequently, while we use the findings of this study to ensure that the content of the messages are clear, unambiguous and accessible, we rely on the overarching principles of BIs in this field to define the specific content. The result is a low-intensity ‘brief’ mHealth intervention consistent with the concept of BI. Evaluating the extent to which the intervention is effective is the intended aim of a formal randomised controlled trial in progress.

The text message intervention is designed to be automated and unidirectional, which aids the ability to provide a cost-effective and scalable service. A limitation of this is the relative lack of personalisation and interactive functionality. This was reflected in feedback from respondents who expressed the positive attributes of being able to interact with the service provider, e.g. texting back and forth with someone, although others indicated some value in a less personalised approach which provided a greater level of assurance regarding privacy. A recent mHealth qualitative study by Ranney et al. (*n* = 20) found that adolescent females (presenting in ED and at high-risk for violence and depressive symptoms) understood that text messages might be automated, but that they should be individually tailored with some two-way communication. Participants said that both automated and as-needed messages (*i.e.* messages that could be requested) would be useful [39]. Another approach is that of Renner and colleagues’ who have explored the idea of people creating their own text messages, which are then delivered at times stipulated by the recipient based on their own drinking habits [40]. While the YourCall text-message intervention did not have formal interactive features due to resource constraints, we included text messages which provide respondents with the free-phone number for the New Zealand Alcohol & Drug Helpline.

This research demonstrates a robust methodology for developing a text message intervention, based on components of Whittaker and colleagues’ model [27]. Our research has built on previous feasibility work [25] to progress the concept of a brief text message intervention for hazardous alcohol use from a hypothetical idea to a fully-developed intervention. A key component of this process is the involvement of the target audience and other stakeholders to provide feedback on the prototype.

An important focus of this research was the creation of culturally-appropriate text messages, to assist with engagement. In New Zealand, where Māori and Pacific peoples experience inequities in the burden of alcohol-related injury outcomes and other alcohol-related harms, it is critical that interventions are developed which are relevant for the diverse realities of Māori and Pacific peoples and are implemented via channels (such as mobile phone) which have the potential to reduce inequalities in access to healthcare services [20, 21].

## Conclusions

We have developed a text message intervention underpinned by established BI evidence and behaviour change theory to reduce harmful drinking among patients admitted following an injury who screen positive for hazardous alcohol use. While text messages remove the interpersonal component of BI, they can be viewed as an approach that distils BI to its core information elements. We employed a formative research process involving feedback from the target audience, service providers, and other key stakeholders to contextualise the content of the intervention and enhance its acceptability and appropriateness for the intervention setting. The next important step is evaluating the effectiveness of the intervention, the objective of a randomised-controlled trial currently in progress.

## Appendix 1: Summary of interviewer guide

The content of this document was developed by the research team as a guide for the interviewers. It was designed to act as a prompt for the interviewers during face-to-face semi-structured interviews with participants, rather than a rigid set of questions. The interviewers were able to be flexible, adapting the questions and asking other questions as needed, depending on the participant and the ideas that emerged during the interview.

### Part One: Exploring views on content of text messages

The following prompts were adapted and asked in relation to each text message:

1. What do you think about this message? (E.g. language and tone.)
2. Does the message make sense to you? Why/why not?
3. Purpose:
  - What do you think the message is trying to tell you?
  - Our idea with this message is to [*add specific purpose of message*]. What are your thoughts about this? Do you think the message achieves the purpose intended?
  - The above were complemented with more general content questions such as:
    - What particular types of information might motivate you to want to make a change?
    - What sorts of messages would work for you?
    - Do you think messages should be different for different groups of people?
    - What groups should we think about having different messages for? (E.g. gender, age, ethnic groups.)

### Part Two: Perceptions about length of intervention and frequency of messages

1. What do you think about the length of this intervention, *i.e.* 4 weeks?
2. How do you feel about getting text messages frequently?
3. What frequency of text messages would work for you?
4. Consider the first week of messages. How do these messages make you feel? Are they too far apart? What do you think about the level of support provided during the first week?

### Part Three: Perceptions about interactivity

1. How could we improve this programme?
2. What other functions would you like to be offered?
3. Do you think the programme should be more interactive? (E.g. should you be able to text for more information or motivational tips? How soon would you want a reply?)

### Part Four: Cultural aspects

[*These questions were adapted depending on the ethnicity of the participant.*]

1. We want to ensure the messages are relevant for Māori participants. How can we do this?
2. What things do you think are important to consider for text messages for Māori people?
3. Do you think it would be useful to have text messages or words in Te Reo Māori?
